# A Comparative Genomic and Phylogenetic Analysis of the Origin and Evolution of the CCN Gene Family

**DOI:** 10.1155/2019/8620878

**Published:** 2019-06-19

**Authors:** Kuan Hu, Yiming Tao, Juanni Li, Zhuang Liu, Xinyan Zhu, Zhiming Wang

**Affiliations:** ^1^Department of Hepatobiliary Surgery, Xiangya Hospital, Central South University, Changsha, Hunan, China; ^2^Department of Pathology, Xiangya Hospital, Central South University, Changsha, Hunan, China; ^3^Department of Reproductive Medicine, Affiliated Hospital of Jining Medical University, Jining, Shandong, China; ^4^Department of Gastroenterology, Shanghai East Hospital, Medicine School of Tongji University, Shanghai, China

## Abstract

CCN gene family members have recently been identified as multifunctional regulators involved in diverse biological functions, especially in vascular and skeletal development. In the present study, a comparative genomic and phylogenetic analysis was performed to show the similarities and differences in structure and function of CCNs from different organisms and to reveal their potential evolutionary relationship. First, CCN homologs of metazoans from different species were identified. Then we made multiple sequence alignments, MEME analysis, and functional sites prediction, which show the highly conserved structural features among CCN metazoans. The phylogenetic tree was further established, and thus CCNs were found undergoing extensive lineage-specific duplication events and lineage-specific expansion during the evolutionary process. Besides, comparative analysis about the genomic organization and chromosomal CCN gene surrounding indicated a clear orthologous relationship among these species counterparts. At last, based on these research results above, a potential evolutionary scenario was generated to overview the origin and evolution of the CCN gene family.

## 1. Introduction

The CCN family consists of six cysteine-rich proteins designated CCN1 to CCN6. CCN as an acronym here represents the first three members of this family that have been discovered: CYR61 (cysteine-rich 61), CTGF (connective tissue growth factor), and NOV (nephroblastoma overexpressed) [1]. These first three identified genes are therefore given the name as CCN1 (CYR61), CCN2 (CTGF), and CCN3 (NOV) successively. CCN1 (CYR61) was first discovered in fibroblasts as an immediate-early gene that can be induced by serum or growth factors [2]. It was the 61st found in CYR family, hence came the name CYR61. CCN2 (CTGF) got its name from being able to increase the mitogenic activity of connective tissue in vivo when it was first known by chance [3]. CCN3 (NOV) was first cloned from a proviral DNA insertion site of MAV1 (myeloblastosis-associated virus type 1), which was termed by people for its role of inducing nephroblastoma in chicken [4]. CCN4 (WISP1, WNT1 inducible signaling pathway protein-1), CCN5 (WISP2, WNT1 inducible signaling pathway protein-2), and CCN6 (WISP3, WNT1 inducible signaling pathway protein-3) used to belong to the WISP gene family, but subsequently researchers classified them into CCN family because of the four highly conserved principal domains they all have in common. In this study, to facilitate the research, we will follow the unified nomenclature to describe all the CCN family members such as CCN1-CCN6 [1].

The CCN family of multifunctional proteins are implicated in numerous vital biological functions, such as cell survival, differentiation, angiogenesis, tumorigenesis, and wound healing [5–7]. Also, this family of proteins has a prototypical common feature in primary structure-a secretory signal peptide at N-terminal followed by four conserved functional domains. They are insulin-like growth factor binding protein-like domain (IGFBP), von Willebrand factor type C repeat domain (VWC), thrombospondin type-1 repeat domain (TSP-1), and the cysteine-knot containing domain (CT). Besides, 38 cysteine residues distributed across the four functional domains were found having significant conservation [8]. The three-dimensional structure of the CCN proteins in Homo sapiens had been well characterized [9]. However, the quaternary structures of CCNs have not yet been elucidated in any species.

For the past decades, researchers have shown growing interest in revealing the expression and functions of the CCN family. CCNs have been reported to exhibit a broad and variable expression in embryonic tissues and adult and showed the pivotal role in either wide variety of biological functions or pathogenesis of diverse diseases. For the biological functions, CCNs have been involved in regulating cell growth, differentiation, proliferation, fundamental biological processes including extracellular matrix remodeling, skeletal development, and chondrogenesis, angiogenesis, and wound repair [8, 10–16]. Besides, the abnormal expression of CCNs participated in organ fibrogenesis, diabetes, retinopathy, and tumorigenesis [13, 14, 17–21].

However, the expression patterns and function of CCNs among different species present both diversity and similarity. To illustrate that, we use 2 CCN family members (CCN1 and CCN2) which have been most extensively studied in the family. Mouse CCN1 was expressed highly in uterine epithelial cells; its high level in early stage embryogenesis plays an indispensable role in the formation of chorioallantoic fusion and placental vessel, which is the critical step of placental development [22, 23]. Mouse CCN1 was also found in chondroblasts. It was proved to participate in the process of osteo/chondrogenesis together with CCN2. Mouse CCN2 exists in many tissues but has the highest concentration in vascular tissues and chondrocytes. Researchers found that CCN2 is required in the process of chondrocyte proliferation and extracellular matrix composition by VEGF, which is inhibited after binding to TSP domain of CCN2 [24].

The Xenopus CCN1, which is mainly located at the cytoplasm of fertilized eggs, turned out to be involved in the regulation of gastrulation through WNT signaling pathway. Interestingly, both CCN1 overexpression and downregulation lead to failure of gastrulation, indicating that optimal CCN1 plays a vital importance role in the process of gastrulation [25]. CCN2 in Xenopus is distributed in somites, notochord, floor plate, and several other tissues. An antidorsalizing effect was reported due to downregulation of CCN2, which is mediated by the inhibition of WNT signals [26].

In zebrafish, CCN1 is present in many tissues such as the dorsal brain, notochord, and otic vesicles, while CCN2 is predominantly expressed in the somites and the notochord, which is partially overlapped with its expression patterns in other vertebrate species [27]. Regarding the function, upregulated CCN2 was found during zebrafish spinal cord regeneration after injury. CCN2 is involved in bridging events initially, thus facilitating regeneration. On the other hand, mutation of CCN2 considerably obstructs this regeneration [28]. Another study about zebrafish indicated that, consistent with CCN2's role in mouse, knockdown of CCN2 gene disrupted the development of notochord [29].

In summary, CCN family members have highly conserved property in their structure. Meanwhile, their expression patterns and function seem to be similar or distinct among different species. So, an interesting question about how they evolve has surfaced. This study aimed to trace the origin of the CCN protein family, deduce the possible evolutionary process preliminarily, and compare the similarities and differences among CCN homologs based on the comparative analysis. We first performed an extensive BLAST to identify CCN homologs among representative organisms from vertebrates to invertebrates, during which functional sites such as transmembrane region and N-glycosylation location were also observed. The phylogenetic analysis was performed based on their genomic sequences. Finally, we got the probable evolutionary scenario of the CCN gene family.

## 2. Materials and Methods

### 2.1. Data Extraction

The amino acid sequence of CCN2 (Genbank number: NP_001892, which is the most extensively studied CCN member in the family) was used as the source to extract all the CCN homologs from different species that are enrolled in this study. The whole process of data extraction is described as follows: type in CCN2 amino acid sequence for the BLASTP searching in the NCBI (http://www.ncbi.nlm.nih.gov/sites/entrez) and Ensembl database (http://www.ensembl.org/Multi/blastview), then choose, and verify the potential CCN homologs in different species. The species enrolled in this study include the following: human (*H. sapiens*), mouse (*M. musculus*), clawed frog (*X. tropicalis*), zebrafish (*D. rerio*), lamprey (*L. japonica*), amphioxus (*B. floridae*), sea squirt (*C. intestinalis*), fruit fly (*D. melanogaster*), Cestoda (*E. multilocularis*), oyster (*C. gigas*), trichina (*T. patagoniensis*), roundworms (*C. elegans*), earthworm (*Lumbricina*), hydra (*H. magnipapillata*), paramecium (*P. caudatum*), and yeast (*S. cerevisiae*). Cross BLAST searching was also performed, respectively, to make sure the identities of sequences that were returned from the database.

### 2.2. Sequence Analysis

We used this sequence information to predict functional domains by searching the SMART database (http://smart.emblheidelberg.de). The domain regions we got further underwent verification by Pfam (http://pfam.sanger.ac.uk/search). MEME (Multiple Expectation Maximization for Motif Elicitation, Version 4.11.4.) (http://meme.sdsc.edu/meme/meme-intro.html) was used as a tool to find out shared motifs and potential binding sites among CCN protein sequences from different species. Besides, TMpred server (http://www.ch.embnet.org/software/TMPRED_form.html) was used to predict the probable transmembrane sequences. Also in the present study, predictions for potential signal peptide and N-glycosylation site were separately performed through SignaIP 4.1 server (http://www.cbs.dtu.dk/services/SignalP/) and NetNGlyc 1.0 (http://www.cbs.dtu.dk/services/NetNGlyc).

### 2.3. Evolutionary Analysis

DNAMAN and ClustalX were applied to perform alignments among interspecies multiple CCN protein sequences. Two algorithms—Neighbor-Joining (NJ) and Maximal Parsimony (MP)—were applied to deduce the phylogenetic analyses by use of the MEGA package (version 7.0). Bootstrap with 1000 iterations was set to reach branch confidence values; P-distance and Pairwise Deletion (to handling missing data) were the Preferences Model/Methods in the phylogenetic analyses. Besides, the surrounding genes next to CCNs in the chromosome were delineated through the Ensembl database and NCBI Map Viewer assemblies (https://www.ncbi.nlm.nih.gov/genome/gdv/). Representative species enrolled are listed as follows:* H. sapiens*,* M. musculus*,* X. tropicalis*,* D. rerio*,* C. intestinalis,* and* D. melanogaster*. The synteny analysis was performed to explore the conservative degree of genomic neighborhoods of CCNs. At last, the corresponding exon structure (exon number, sizes, and transcriptional orientations) was acquired from NCBI and Ensembl databases and then compared with each functional domain of CCN protein sequences.

## 3. Results

### 3.1. Amino Acid Sequence Identification

**Figure 1:**
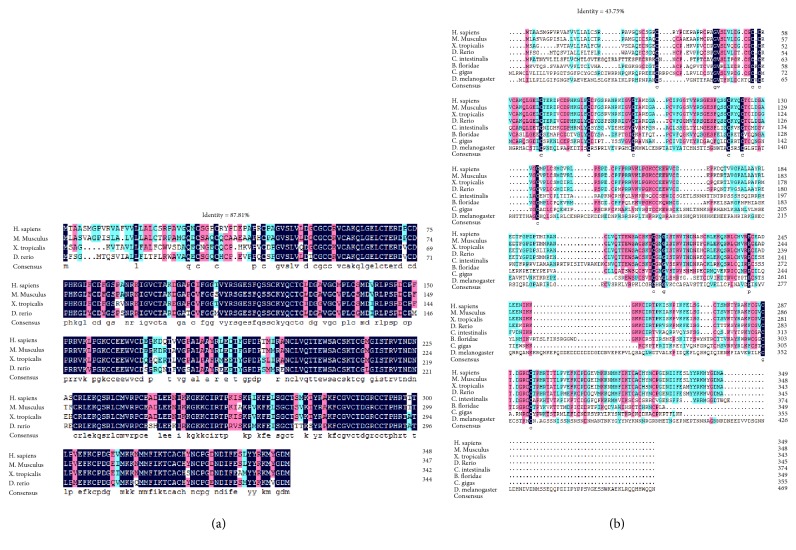
*Multiple sequence alignment of CCN2 proteins in various species*. The alignment was established by loading the sequences to DNAMAN and ClustalX. (a) Amino acid alignment for vertebrates (*H. sapiens*,* M. musculus*,* X. tropicalis*, and* D. rerio*); (b) amino acid alignment containing vertebrates and invertebrates (*H. sapiens*,* M. musculus*,* X. tropicalis*,* D. rerio*,* C. intestinalis*,* B. floridae*,* C. gigas*, and* D. melanogaster*).

We used the human CCN2 amino acid as the queries in BLASTP, Genbank, PubMed, and Ensembl database for searching CCN homologs/family members among all kinds of species. CCN homologs/family members from representative species (*H. sapiens*,* M. musculus*,* X. tropicalis*,* D. rerio*,* L. japonica*,* B. floridae*,* C. intestinalis*,* D. melanogaster*,* E. multilocularis,* and* C. gigas*) were chosen and showed in Table 1 together with their accession number and necessary structure information. As Table 1 reveals, 6 CCN homologs were found in mammals including human and mouse, which were reported as six members of CCN family in human (CCN1-CCN6). Likewise, six homologs were identified in clawed frog, 9 in zebrafish, 4 in lamprey, and 5 in ascidian. Only one homolog was verified in amphioxus, cestode, oyster, and fruit fly. Interestingly, no homolog was found in relatively more primitive organisms (we searched in* trichina*,* elegans*,* Lumbricina, Hydra*,* Paramecium, and Saccharomyces*). To further explore the relevance among these amino acid sequences from different species, we did multiple sequence alignment of CCN2 proteins in vertebrates (human, mouse, clawed frog, and zebrafish) and in all the species from Table 1. Strikingly, high identity was delineated in these two alignments (87.81% among chordates; 43.75% for all) (Figure 1).

### 3.2. Structural Organizations of CCNs

As the most widely studied protein in CCN family members, human CCN2 consists of a 26-amino acid (aa) signal peptide and four domains: IGFBP (71aa), VWC (64aa), TSP (44aa), and CT domain (70aa) (Table 1). We found that vertebrate CCN2 not only have a similar length of the amino acid sequence but also retain the typical structure we described above: one signal peptide followed by four specific domains; each domain had little amino acid difference in length among different vertebrate species. Furthermore, not limited to CCN2s, the typical structure existed in common in all the CCN members from various species when we performed SMART to analyze the other CCN family members/homologs. However, some of the domains were missing. All the CCN5s lacked CT domain in vertebrates; CCN2-like lose the VWC domain in amphibian; no signal peptides, IGFBP, and VWC domain existed in some ascidians. Understanding the universalities and differences in protein structure could help us better explore the function of CCNs in different species.

**Figure 2:**
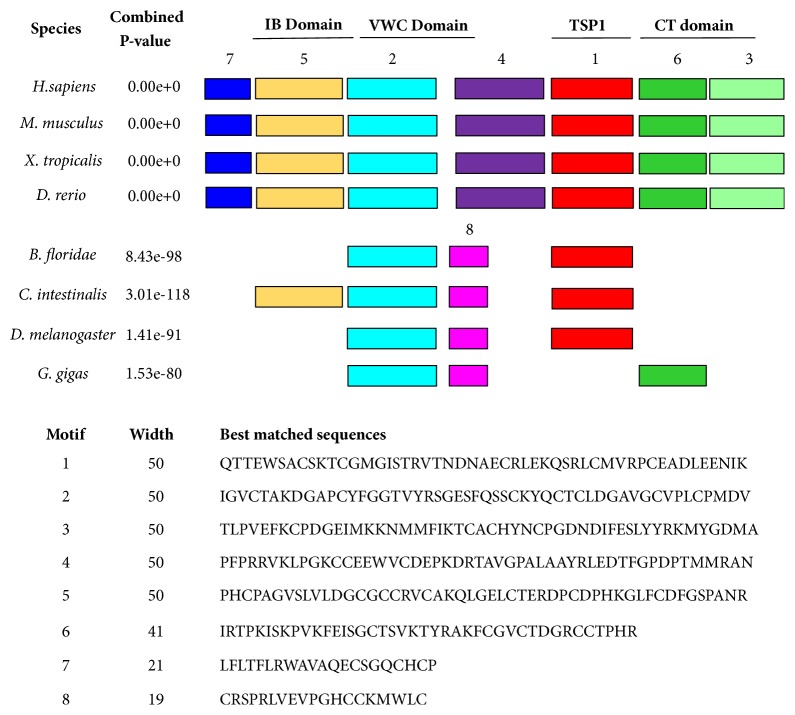
*MEME SUITE showed the structural organization of CCN family members from representative species*. Eight motifs were returned from MEME analysis, and different colors represent different motifs. The best matches (consensus sequences) are listed at the bottom of the figure for all the motifs.

Because most of the CCNs have similar domains and the roles of these functional domains have not been well characterized, we used MEME server to further research smaller functional motifs of CCNs. As Figure 2 exhibits, eight representative species were enrolled, and eight motifs were identified by MEME server. Eight motifs showed extremely high consistency in vertebrates (*H. sapiens, M. musculus, X. tropicalis, and D. rerio)*. Among these eight motifs, motif five was detected in IGFBP domain, motif 2 and part of motif 4 were detected in WVC domain, motif 1 was detected in TSP1 domain, and motifs 3 and 6 were detected in CT domain. However, when we came to the lower organisms after comparing with vertebrates, things started to become different. Motifs gaining and losing happened in these species, respectively, according to Figure 2. Only three conserved motifs were found in* B. floridae *and* D. melanogaster*, five were found in* C. intestinalis*, and three were found in* G. gigas*.

### 3.3. N-Glycosylation Site, the Transmembrane Region of CCNs

The changes of glycosylation profile in some glycoproteins (such as CCN3) are related to various physiological and pathological processes, ranging from cell migration, differentiation to tumor invasion [30].* N*-glycoproteins as a highly regulated process also play an essential role in growth, differentiation, and tumor cell metastasis. In the present study, we recorded the* N*-glycoproteins sites among different CCNs (as presented in Table 1), which showed great diversity [31]. There are only a few CCN members which undergo glycosylation modification in each species. For instance, CCN 2,3,4 are only glycosylated in human and claw frog, CCN 3,4 only in mouse, and CCN 1,4,5 only in zebrafish; no CCN is glycosylated in amphioxus.

Furthermore, a significant difference has also been found in the distribution and number of glycosylation sites in different species. In general, it tends to have less than three glycosylated sites in vertebrates, but, for the invertebrates, there are more glycosylated sites that can reach 7 (such as fruit fly). Additionally, we observed that most of the glycosylated sites are dispersedly distributed in 3 well-conserved domains of CCN family (IGFBP, VWC, and CT domains) except TSP1 domain.

We then focused on the transmembrane region of each CCN. As shown in Table 1, the majority of CCNs contain at least one transmembrane region located at the beginning of N-terminal, which is also the location of signal peptide, while there are two or three transmembrane regions in some vertebrates in CCN4, 5, 6, revealing the heterogeneity as well as the conservation of the transmembrane region.

### 3.4. CCN Gene Family Undergoes Lineage-Specific Expansion

**Figure 3:**
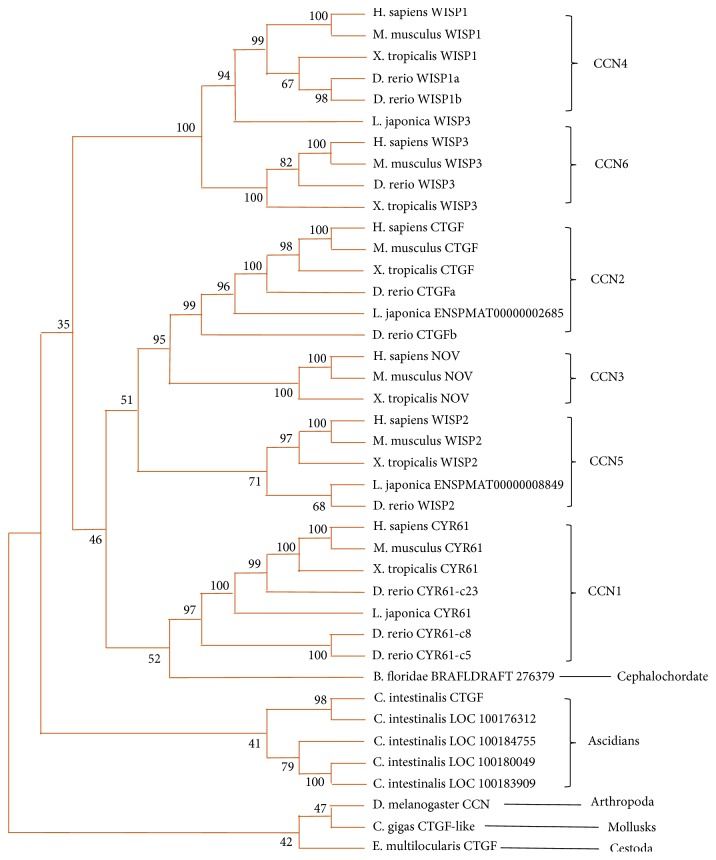
*The establishment of the phylogenetic tree of CCNs*. The phylogenetic tree was established by the neighbor-joining method with the alignment. Most of the homologs in the figure are described as “species name + gene name,” despite the exception that Ascidian CCNs are shown as “species name + gene symbol” to avoid gene name repetition. The brace on the right side sorts the homologs according to CCN family member individually. The bootstrap percentage was presented near the location where branches cluster together.

Next, we were curious if there is a potential evolutionary clue to the CCN gene family. For this purpose, both NJ and MP algorithms were done after all the CCNs' amino acid sequences were input into Mega 7 to draw the phylogenetic trees. Considering the consequence of NJ and MP algorithms is almost the same, and a single phylogenetic tree was constructed after merging them (Figure 3). As shown in Figure 3, the first conclusion we could get is that flatworm CCN (*E. multilocularis*) might be the ancestor of the CCN gene family among all species because they were found located at the base. Then the clade of flatworm CCN orthologs branched externally to form arthropods and mollusk CCNs, which further branched externally to ascidian (*C. intestinalis*) CCN homologs. After that, the processes of evolution become entirely different when it comes to vertebrates. The clade containing flatworm, arthropods, and mollusk CCN sequences split into two clades containing vertebrate CCN1/2/3/5 and CCN4/6, respectively. These two clades also split into subclades containing individual CCN1, CCN2, CCN3, and CCN5 orthologs and CCN4 and CCN6 orthologs. The kinship among the six clades (CCN1 to CCN6) was easy to tell from the tree: CCN 1, 2, 3, 5 were more similar to each other. CCN 4 and 6 stayed close and alienated to CCN 1,2,3,5 relatively. Furthermore, after looking into each clade, we found that the four kinds of vertebrates are orthologs and closely related to each other, which is consistent with their high conservation. These results taken together suggest that CCN genes have undergone lineage-specific expansion, where a single proto-ortholog to CCN duplicated to create current CCN family members.

It was also worth mentioning the difference that the species (zebrafish, lamprey, and ascidian, etc.) that live in water have different CCN members, while vertebrates that live on land consistently have all the six of CCNs. It might be the consequence of potent selection pressures given by living environments during evolution.

### 3.5. Gene and Genomic Organization

**Figure 4:**
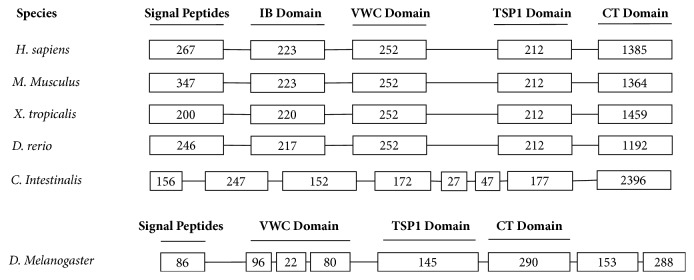
*Schematic diagram of the exon organization of CCN2 in representative species*. Species names were listed on the left. In the diagram each box stands for an exon, and the number inside the box represents the base-pairs number of each exon. Exon organization was then aligned with the domains of the corresponding sequence.

In order to explore the evolutionary heritage of CCNs, we looked at their genomic organization among representative species (Figure 4). Intriguingly, the genomic structural comparison of CCNs shared extreme similarity among all the vertebrate species enrolled: same exons numbers (n=5) and very close size for every exon. The relatively restricted genomic organization implied the existence of a potent selective pressure in the process of vertebrate evolution. Five exons corresponded to five parts of the structure: one signal peptide and four highly conserved domains (IGFBP domain, VWC domain, TSP1 domain, and CT domain), respectively.

Furthermore, for the two exons that encode VWC and TSP1 domain, it is striking to find that they have undoubtedly the same size (252bp and 212bp) in all the vertebrates examined, suggesting that their structures and functions have been well preserved by evolutionary constraints. However, variations appeared when we took a look at the CCNs in* C. intestinalis* and* D. melanogaster*. They have limited similar pattern compared to vertebrate species, while there are in total eight exons instead of five, which size showed the apparent difference from any other orthologs. It had no more one-to-one correspondence between exons and function domains—actually, some extra exons are encoding unknown insertion sequences beyond the four conservative domains we mentioned above, suggesting that exons restitching happened in the process of evolution, which contributes to the CCNs evolution from ancient arthropods/ascidians to chordate.

**Figure 5:**
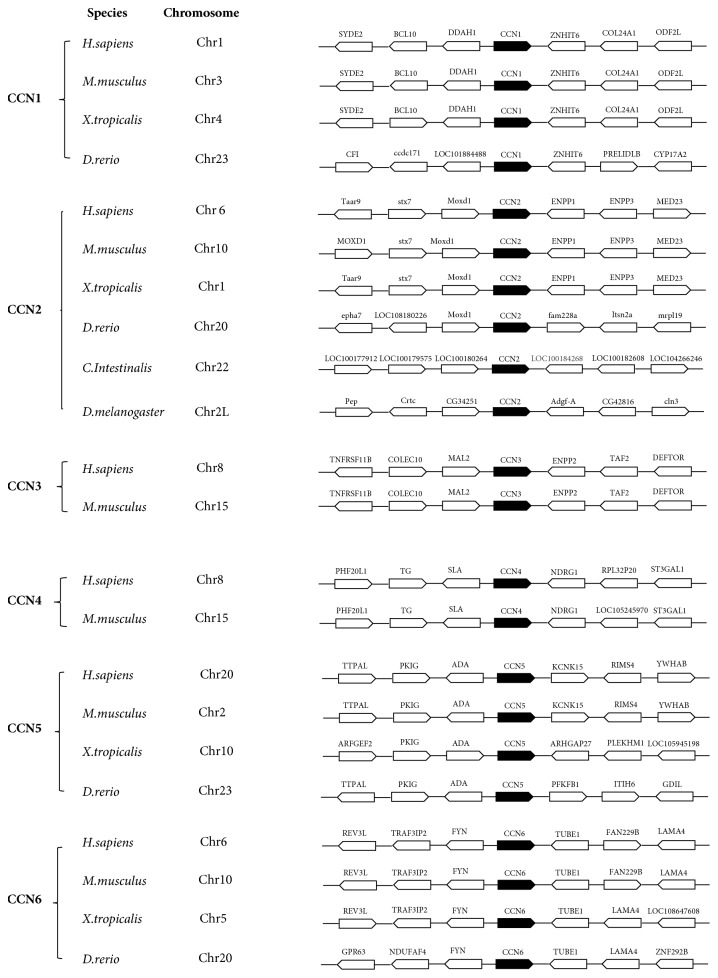
*Chromosomal disposition of CCNs showed conserved synteny in mammals and frog*. The genomic neighborhood surroundings of CCNs in single chromosome were observed. The black pentagon in the figure represents the CCN gene, while the white pentagon represents surrounding genes next to the CCN gene. Arrow of each pentagon represents the transcription orientation of each gene.

To further illustrate the genomic sequences and neighborhood surrounding, the neighboring genes of CCNs were observed in mammals, frogs, fish, ascidians, and fruit flies (Figure 5). Here we discussed CCN2 as a typical example in the CCNs family first. CCN2 are located on a single chromosome in all species from mammal to fruit fly, revealing that partial gene duplication is the potential way for CCN genes expansion after being separated to different lineages independently. Furthermore, as shown in Figure 5, mammal and frog CCN2 genes enrolled share a conspicuously analogous genomic neighborhood surrounding (almost surrounded by the same genes). However, in lower organisms (zebrafish, ascidian, and fruit fly), the chromosomal location and surrounding become a little different. Consistent result was got for the remaining CCN family members presented on Figure 5. CCN1, 3, 4, 5, 6 showed highly conserved genomic neighborhood surrounding in chordates. However, no similarities of genomic neighborhood surrounding were found among CCN family members; they locate in different chromosomes and have different surrounding genes. These results demonstrated the existence of a potent level of conserved synteny, which not only leads to limited chromosomal rearrangements with known karyotypes [32], but also indicates a clear orthologous relationship among these species counterparts.

## 4. Discussion

### 4.1. The Highly Conserved Structure of CCN Proteins among Species Is the Basis of the Functional Similarities

The CCN family of proteins is an ancient, highly conserved family. To chase their origin and evolution, we used the protein sequence of human CCN to track down all the CCN homologs in species from higher organism to lower organisms. Based on the visualized result from sequence analysis, which showed a quite high identity (43.75%) in enrolled species and even higher identity (87.81%) in vertebrates, this identity was most likely to happen among homologs and could be the structural basis for their similar functions [33].

The structure of CCN proteins was shown highly conserved among various species (especially in vertebrates) according to BLAST, SMART, and motif analysis, despite the very few absences/alterations of the specific domain in certain species we mentioned above. CCN members are multimodular mosaic proteins consisting of four highly conserved modules: IGFBP domain, VWC domain, TSP-1 domain, and CT domain. We can find many genes that share one or two domains with CCNs, but the order and combination of the four domains are specific and unique to the CCN family.

SMART and MEME motif analysis showed that TSP and VWC domains, which can be found in lower animals such as fruit flies, are relatively more conservative, while IGFBP and CT domains only keep the highly conservative property in vertebrates. They abandon or lose some part of motifs in lower animals. Thus, it is possible that IGFBP and CT domains are required for the emergence of vertebrates under selection pressure and essential for maintaining some functions for vertebrates. The biological activities that CCN proteins possess are entirely consistent with the functions of each module or interacted functions among modules. IGFBP domain can stimulate cell proliferation through the JNK pathway. This domain of CCN3, 4, 5 was also reported involving tumorigenesis, especially in breast cancer [34]. VWC domain only has one copy in CCN proteins; its primary function is about regulating bone morphogenic proteins and TGF-*β*, thus affecting organ growth and development as well as skeletal formation [35]. TSP domain of the CCN family is known to have a vital role in extracellular matrix accumulation and angiogenesis through interacting with sulfated glycoconjugates and integrins [16]. CT domain, the last of the four conserved domains, is one of the most critical domains because of its role in CCN functions preservation, protein-protein interactions, and connection with other domains [26, 36].

On the other hand, there was also the structural heterogeneity in the modular configuration that contributes to diversity in CCN functions. So the absence of certain modules in some CCN members (like CCN5) or some invertebrate species (like* C. intestinalis* or* D. melanogaster*) could cause massive diversity in their functions. It was an excellent example to illustrate the fact that CCN3 knockout mice without the VWC domain can lead to deformed cardiac and skeletal development [37]. Up to now, most of the published papers about CCNs have been focused on vertebrates. However, little is known about the functions and related structural features of CCNs in the lower organisms. Here we used the structural homology as a bridge to link CCNs from various species together and thereby preliminarily made some functions of CCNs predictable. So, the study might give us a better understanding of the relationship between the functions and structure of CCNs among all the species. Besides, it might help us understand the certain functions of CCNs and explore their potential role that has never been found by now.

### 4.2. Hypothetic Evolutionary Relationships of CCN Gene Family

**Figure 6:**
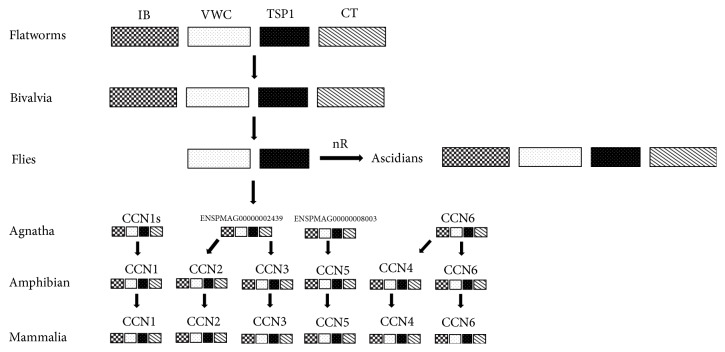
*Potential evolutionary relationships of the CCN gene family*. A diagram was drawn to illustrate the potential evolutionary scenario among the CCN gene family. Rectangles with different colors stand for the different domains. CCN is likely to originate from the gene with a relatively primitive formation in* E. multilocularis *(flatworm). Arrow indicates the evolutionary process from ancient CCNs to present-day CCNs, during which massive homologs are produced due to numerous rounds (nR) of extensive gene duplication events and lineage-specific expansion.

A simple model had been made to briefly demonstrate the hypothetic evolutionary scenario of CCN genes based on the analyses of comparative genome and phylogenetics (Figure 6). After tracing CCN genes from higher organisms to lower organisms (such as paramecium or yeast), we proposed that the origin of the CCN gene could date back to the emergence of flatworms. During the evolutionary process, CCN gene of arthropods lost the IGFBP and CT domains because of exon restitching events under the pressure of selection. In the relatively complex aquatic environment, CCN genes of ascidians undergo extensive lineage-specific duplication so that homologs are therefore produced.

Thereafter, the branching patterns of CCNs become clearer in chordate lineage. During the evolutionary process, a single proto-ortholog to CCN undergoes multiduplication events to create two ancestor genes as ancestor CCN1/2/3/5 and CCN4/6. Both CCNs genes led to creation of nowadays CCN family members from CCN1 to CCN6. Then, ancestor CCN1/2/3/5 produce CCN1, CCN5, and relative ancestor gene CCN2/3 firstly; then ancestor CCN further duplicates to CCN2 and CCN3. Four CCNs sequences observed in lamprey are good evidence to support this deduction event because CCN4/6 and CCN2/3 in lamprey have still not yet undergone duplication. By knowing these, the hypothetic evolutionary process shows a clear orthologous relationship and it is helpful to understand the origin of present-day CCNs.

In summary, comparative study on the features (structure and function) of CCNs among different species can serve as the basis and evidence of extensive gene duplication events and lineage-specific expansion during the long-term evolution. However, to be rigorous, more specific experiments are required in the future to confirm these features we observed.

## Figures and Tables

**Table 1:** CCN genes from representative organisms.

Class	Species	name	accession number	Protein length in amino acid(aa)	Signal Peptides	Position of IGFBP Domain	Position of VWC Domain	Position of TSP1 Domain	Position of CT Domain	Trans-membrane	Predicted positions of N-glycosylation
Mammals	*Homo sapiens *	CCN1	NP_001545	349	1-24	24 - 93	100 - 163	231 - 273	291 - 360	1-23	-
		CCN2	CAG46534	353	1-26	27 - 97	103 - 166	200 - 243	261 - 330	1-18	28
		CCN3	NP_002505	362	1-31	33 - 104	110 - 173	207 - 250	269 - 338	14-32	97
		CCN4	NP_003873	372	1-22	47 - 117	123 - 185	217 - 260	278 - 347	7-25,110-128,216-241	86,143
		CCN5	NP_001310299	253	1-23	24 - 93	100 - 163	194 - 238	-	199-217	-
		CCN6	NP_937882	377	1-33	64 - 134	140 - 197	229 - 271	291 - 360	21-37,158-176	196,326
	*Mus musculus*	CCN1	NP_034646	379	1-24	24 - 93	100 - 163	229 - 271	289 - 358	1-23	-
		CCN2	NP_034347	348	1-25	26 - 96	102 - 165	199 - 242	260 - 329	1-17	-
		CCN3	NP_035060	354	1-25	27 - 98	104 - 167	204 - 247	266 - 335	10-27	91,277
		CCN4	NP_061353	367	1-22	49 - 117	123 - 185	217 - 260	278 - 347	4-25,110-128	86,143,284
		CCN5	NP_058569	251	1-23	24 - 93	100 - 163	195 - 239	-	6-26,200-218	-
		CCN6	NP_001120848	354	1-19	46 - 116	122 - 179	211 - 253	273 - 342	1-19,140-157	-

Amphibian	*Xenopus tropicalis*	CCN1	NP_001098984	375	1-24	24 - 93	100 - 163	226 - 268	286 - 355	3-24	-
		CCN2	NP_001015042	343	1-21	22 - 91	97 - 160	194 - 237	255 - 324	1-18	-
		CCN3	XP_002938019	344	1-18	19 - 89	95 - 158	191 - 234	253 - 322	1-19	264
		CCN4	XP_002939562	358	1-19	39 - 109	115 - 177	209 - 251	269 - 338	3-22,102-125,318-336	21,78,135
		CCN5	NP_001011237	312	1-23	24 - 92	98 - 161	190 - 234	-	4-26,195-213	-
		CCN6	XP_002936979	368	-	62 - 131	137 - 195	225 - 267	287 - 356	-	-

Bony fishes	*Danio rerio*	CCN1-c23	NP_001001826	369	1-19	19 - 88	95 - 158	219 - 261	279 - 348	1-17	275
		CCN1-c5	NP_001017653	383	1-28	28 - 97	104 - 167	229 - 272	290 - 362	6-29	-
		CCN1-c8	NP_001074456	373	1-26	30 - 99	106 - 169	223 - 266	281 - 352	11-29	333
		CCN2a	NP_001015041	345	1-23	24 - 93	99 - 162	196 - 239	257 - 326	2-18	-
		CCN2b	NP_001096043	347	1-22	23 - 92	98 - 161	196 - 238	256 - 325	1-22	-
		CCN4a	NP_001159702	360	1-19	44 - 114	120 - 182	212 - 255	273 - 342	1-18,325-344	41,155,279
		CCN4b	NP_001107100	356	1-17	39 - 109	115 - 177	206 - 249	267 - 336	1-19	21,273
		CCN5	NP_001186030	344	1-28	29 - 98	104 - 167	203 - 246	265 - 334	13-31	228
		CCN6	NP_001159703	338	1-20	32 - 101	107 - 146	195 - 237	257 - 326	1-18,125-143	-

Agnatha	*Lampetra japonica*	CCN1I2	ENSPMAG00000002981	387	-	24-94	101-167	235-278	298-366	5-23	-
		CCN6	ENSPMAG00000001225	370	-	46-116	122-185	221-263	281-350	286-306	-
		ENSPMAG00000002439	ENSPMAG00000002439	370	-	28-97	103-162	217-259	377-346	-	-
		ENSPMAG00000008003	ENSPMAG00000008003	174	-	-	3--67	128-171	-	-	-

Leptocardii	*Amphioxus B. floridae*	BRAFLDRAFT_276379	XP_002600149	349	1-21	27 - 95	101 - 164	200 - 242	275 - 347	3-22,198-217	-

Ascidian	*Ciona intestinalis*	LOC100181891	XP_002127157	374	1-23	33 - 101	107 - 171	227 - 270	288 - 356	3-21	179,180
		LOC100176312	XP_002131922	401	-	90 - 152	158 - 220	252 - 295	313 - 380	13-29,45-64	-
		LOC100180049	XP_018669063	428	-	26 - 96	102 - 166	274 - 317	335 - 406	5-27	-
		LOC100183909	XP_002130743	419	-	22 - 92	98 - 162	267 - 309	326 - 396	10-30	-
		LOC100184755	XP_002130917	207	-	-	-	59 - 99	118 - 188	25-46	-

Flies	*Drosophila melanogaster *	CCN	NP_730294	470	1-21	-	41 - 107	121 - 164	-	1-17	22,40,45,109, 120,126,365

Cestoda	*Echinococcus multilocularis*	CCN2	CDI98339	-	1 22	-	-	100-143	192-264	-	-

Bivalvia	*Crassostrea gigas*	CCN2-like	XP_011420353	355	1 19	42 - 109	115 - 179	218 - 259	279 - 348	4-25	168,183,187, 212,259,288

“-” means “absence.”

## Data Availability

The data used to support the findings of this study are included within the article.
